# Role of thyroid dysfunction in long-term psychological prognosis of sepsis

**DOI:** 10.3389/fpsyt.2025.1699248

**Published:** 2025-12-17

**Authors:** Enfang Zhao, Chunhua Hu, Wenqing Jia, Tingyuan Zhang, Huanzhang Shao

**Affiliations:** 1 Department of Critical Care Medicine, Henan Key Laboratory for Critical Care Medicine, Zhengzhou Key Laboratory for Critical Care Medicine, Henan Provincial People’s Hospital, Zhengzhou, Henan, China; 2 Zhengzhou University People’s Hospital, Henan University People’s Hospital, Zhengzhou, Henan, China

**Keywords:** anxiety, depression, post-traumatic stress disorder, sepsis, thyroid dysfunction

## Abstract

**Background:**

After recovery from sepsis, approximately 10%-50% of patients develop long-term psychological complications such as anxiety, depression, and post-traumatic stress disorder, yet predictive indicators for these complications remain unclear. Emerging evidence suggests that thyroid function may hold prognostic value for sepsis itself. Building on this evidence, the present study aims to investigate the impact of baseline peripheral thyroid indicators on long-term psychological outcomes (within 28 weeks) in sepsis patients.

**Methods:**

A consecutive sample of 814 sepsis patients was included. Baseline data including demographic characteristics, thyroid function indices, and peripheral inflammatory markers were collected, and psychological outcomes within 28 weeks of follow-up (i.e., anxiety, depression, and post-traumatic stress disorder) were evaluated. ROC analysis, Kaplan-Meier survival analysis, and multivariate COX regression were employed for analysis.

**Results:**

(1) Higher levels of peripheral TT3, FT3, TT4, and FT4 at baseline were correlated with a reduced risk of poor psychological outcomes within 28 weeks. Sepsis-induced hypothyroidism was associated with an increased risk of poor psychological outcomes within 28 weeks. (2) These associations appeared to be more pronounced in elderly patients. (3) Peripheral TSH levels showed no such predictive value; similarly, “low-normal thyroid function” (defined as relatively high peripheral TSH within the normal range) also lacked predictive value. (4) A negative monotonic relationship was observed between baseline peripheral thyroid hormones and peripheral levels of tumor necrosis factor-α, interleukin-6, and interleukin-8.

**Conclusion:**

Diminished thyroid function may be associated with relatively poor long-term psychological outcomes (within 28 weeks) in sepsis patients, possibly more so in the elderly. Given the observed association between thyroid hormones and peripheral inflammatory factors, this potential prognostic relationship may be partially mediated by inflammatory mechanisms. However, this remains a preliminary speculation and requires further validation.

## 1 Introduction

According to the Sepsis-3 criteria, sepsis is defined as life-threatening organ dysfunction caused by a dysregulated host response to infection (1). Globally, more than 19 million people are affected by this disease each year (2). In both the United States and China, there are millions of new patients every year (3, 4). More seriously, the latest mortality data shows that approximately 5.3 million people around the world die from sepsis each year, with a mortality rate as high as 30% to 50% (2).

Given the severe physical impact of sepsis, many survivors develop long-term psychological sequelae. Previous studies have shown that approximately 10% to 50% of patients may suffer from anxiety, depression, or post-traumatic stress disorder (PTSD) even after recovery from the physical manifestations of sepsis (5, 6). Notably, several factors may contribute to or influence the development of these psychological complications, such as age, pre-existing cognitive function, and cumulative use of specific medications in the intensive care unit (e.g., dobutamine, haloperidol) (6). These psychological sequelae significantly impair patients’ quality of life, manifested as persistent emotional distress, reduced self-care ability, and difficulty returning to work or daily activities (7). They often further increase healthcare resource utilization and economic burden, characterized by more frequent outpatient follow-ups, psychological counseling, and even rehospitalization (8). Currently, understanding of the predictive indicators and underlying mechanisms of sepsis-related psychological complications remains insufficient, particularly whether pathophysiological abnormalities during sepsis can serve as early warning signals for such psychological complications. This question remains poorly explored.

It is currently known that many patients with sepsis may experience abnormal thyroid function, mainly manifested as decreased levels of triiodothyronine (T3) and thyroxine (T4), while the serum level of thyroid-stimulating hormone (TSH) may be within the normal range or slightly decreased (9, 10). The underlying mechanisms likely include hypothalamic-pituitary-thyroid axis dysregulation, impaired thyroid hormone synthesis and metabolism, reduced energy metabolism, insufficient thyroid tissue perfusion, and potential effects of drugs such as glucocorticoids and antibiotics (9, 10).

In sepsis patients, thyroid function may aid in assessing disease severity and prognosis, and further guide clinical management. Three observational studies and one Mendelian randomization study have indicated that decreased thyroid hormone levels may be a marker of poor prognosis in sepsis patients, such as an increased risk of short-term mortality (11–14). Meanwhile, there is a complex association between thyroid function and mental status. It is well established that patients with hyperthyroidism (overactive thyroid) often present with symptoms such as anxiety, irritability, and emotional lability, while those with hypothyroidism (underactive thyroid) tend to exhibit manifestations including depression, fatigue, and cognitive impairment (15, 16). Notably, in the context of sepsis, the systemic inflammatory response (which is known to disrupt both thyroid hormone metabolism and central nervous system function) may further strengthen this association (17, 18). This is because inflammatory factors can simultaneously reduce thyroid hormone synthesis and induce neuroinflammation linked to psychological disorders. Therefore, the present study hypothesizes that there is a significant association between thyroid function and mental status in sepsis patients, and that thyroid function during sepsis may predict long-term psychological outcomes in this population. However, this hypothesis still requires verification through research.

Accordingly, this study consecutively enrolled 814 sepsis patients and comprehensively analyzed the associations between acute-phase thyroid parameters (T3, T4, TSH), hypothyroidism, and the development of anxiety, depression, and PTSD within 28 weeks of hospital discharge. The aim of this study was to preliminarily explore the potential predictive value of acute-phase thyroid function abnormalities for long-term psychological outcomes in these patients. The findings of this study may provide a novel and accessible biomarker for screening sepsis patients at high risk of long-term psychological disorders, and offer insights into identifying potential therapeutic targets for improving psychological outcomes in this population.

## 2 Materials and methods

### 2.1 Ethical standards

The study was approved by the Medical Ethics Committee of Henan Provincial People’s Hospital. The entire process was conducted in accordance with the standards of the Declaration of Helsinki of the World Medical Association.

### 2.2 Subjects

From January 1, 2018, to December 31, 2022, consecutive sepsis patients who met the inclusion criteria were enrolled from the Department of Critical Care Medicine, Henan Provincial People’s Hospital. The inclusion criteria were as follows:

1. Meeting the diagnostic criteria of the Third International Consensus Definitions for Sepsis and Septic Shock (Sepsis-3) published in 2016 (19). Specifically, patients had confirmed or suspected infection (which could be identified by clinical manifestations, laboratory tests, and imaging examinations) and presented with organ dysfunction, as defined by a Sequential Organ Failure Assessment (SOFA) score of ≥ 2.
2. Aged ≥ 18 years.
3. Survived the current episode of sepsis and were discharged from the hospital.
4. No history of thyroid disease and no thyroid nodules detected during the current admission examination.
5. No history of hormone therapy, blood transfusion, or use of drugs that interfere with thyroid hormone metabolism within 12 months prior to the current hospitalization.
6. No history of anxiety, depression, or other mental disorders.
7. No history of mild cognitive impairment, Alzheimer’s disease, or other cognitive disorders.
8. Not pregnant and no malignant tumors.
9. Chronic diseases such as hypertension, coronary heart disease (CHD), and type 2 diabetes mellitus (T2DM) were in a stable stage or well-controlled.
10. Provided informed consent to participate in this study, including a 28-week follow-up period.

It should be emphasized that during the follow-up period, any patient who was lost to follow-up, died, received hormone therapy, underwent blood transfusion, used drugs interfering with thyroid hormone metabolism, became pregnant, developed a malignant tumor, or experienced acute exacerbation of chronic diseases (e.g., hypertensive crisis, acute myocardial infarction, diabetic ketoacidosis, etc.) was excluded from this study.

### 2.3 Baseline data

In this study, the baseline was defined as the time point when the patient was in the acute phase of sepsis, hospitalized for treatment, and enrolled in this study.

Basic characteristics, thyroid function, and peripheral inflammation of the patients at baseline were collected from their medical records.

Basic characteristics included gender, age, history of smoking, history of drinking, history of hypertension, history of CHD, history of T2DM, heart rate, body temperature, mean arterial pressure (MAP), peripheral white blood cell (WBC), peripheral hemoglobin, peripheral platelet, peripheral creatinine, peripheral lactate, SOFA score, simplified acute physiology score II (SAPS II) score. Thyroid function included peripheral total triiodothyronine (TT3), free triiodothyronine (FT3), total thyroxine (TT4), free thyroxine (FT4), and TSH. Peripheral inflammatory markers included tumor necrosis factor-α (TNF-α), interleukin-6, and interleukin-8.

History of smoking was defined as regularly smoking tobacco continuously or cumulatively for more than 12 months in one’s lifetime. History of drinking was defined as regularly drinking alcohol continuously or cumulatively for more than 12 months in one’s lifetime. History of chronic diseases was determined by diagnosis and prior treatment in the medical records. All laboratory indicators were tested by the laboratory of Henan Provincial People’s Hospital and quality control was also carried out by this laboratory.

The SOFA score, ranging from 0 to 24, assesses organ dysfunction in patients, covering respiratory, circulatory, liver, kidney, and central nervous systems. A higher score indicates worse prognosis and more severe organ failure (20). The SAPS II score, from 0 to 163, assesses illness severity and predicts mortality risk in the critically ill, incorporating 12 physiological variables, age, hospitalization type, and three chronic diseases. A higher score means more severe illness and poorer prognosis (21).

The normal ranges for peripheral TT3, FT3, TT4, FT4, and TSH were 1.8-2.9 nmol/l, 2.0-6.6 pmol/l, 65–155 nmol/l, 10.3-31.0 pmol/l, and 0.3-5.0 mU/l respectively.

Hypothyroidism caused by sepsis referred to a decrease in FT3 and FT4 levels, while TSH was not limited. Since different levels of TSH within the normal range may have different biological effects, this study divided traditional normal thyroid function into “strict normal thyroid function” and “low normal thyroid function” (22). The former had TSH levels between 0.3 and 2.5 mU/l, and the latter had TSH levels between 2.5 and 5.0 mU/l. Both had normal FT3 and FT4 levels.

### 2.4 Follow-up

Each patient was followed up for 28 weeks starting from the time of discharge. To exclude pre-existing psychological disorders (per inclusion criteria), baseline assessments for anxiety, depression, and PTSD were conducted during hospitalization; a second assessment was performed at the 28th week of follow-up.

The follow-up duration of this study was set based on preliminary observations from pilot studies: psychological issues in sepsis patients mostly occur within 2 to 6 months after hospital discharge. The current follow-up period not only is expected to fully cover the critical onset and stabilization stages of psychological complications but also minimizes the total follow-up duration as much as possible.

Psychological status assessments were only conducted at baseline and the end of follow-up, and no thyroid function tests were performed during the follow-up period. The reasons included the following: repeated tests could easily increase patients’ psychological burden and reduce compliance, thereby affecting the accuracy and completeness of the results; meanwhile, the available data were sufficient to meet the study objective of exploring “the association between baseline thyroid function and long-term psychological prognosis after sepsis”.

It should be emphasized that the assessment at the end of follow-up evaluated the most severe psychological status of patients throughout the entire follow-up period, rather than their psychological status at the specific time point of follow-up end. Additionally, researchers roughly estimated the potential onset time of psychological issues in each patient based on the occurrence time of symptoms specific to anxiety (excessive worry, panic attacks, restlessness), depression (persistent low mood, anhedonia, suicidal ideation), and post-traumatic stress disorder (PTSD, flashbacks, avoidance behaviors, hypervigilance).

Anxiety and depression were evaluated by the Hospital Anxiety and Depression Scale (HADS). It consists of an anxiety subscale (7 items) and a depression subscale (7 items), with respective total scores calculated. Scores of 0–7 indicate normal, 8–10 mild, 11–14 moderate, and 15–21 severe (23).

PTSD was evaluated using the Clinician-Administered PTSD Scale (CAPS), the “gold standard”. It has 17 symptom items scored from frequency, intensity, and distress level (0–4 each). Item score is the sum (0 - 12). Total scale score is 0 - 192. 50+ diagnoses PTSD. Mild: 50 - 60, moderate: 61 - 100, severe: 101 - 130, extremely severe: 131–192 (24).

Since psychological prognosis assessment involves a certain degree of subjectivity, a single-blind design was adopted for this assessment. Specifically, researchers were kept unaware of patients’ baseline data. Two researchers conducted the assessments independently and simultaneously, with each documenting their results separately.

### 2.5 Statistical analysis

After the psychological prognosis assessment, data from the two researchers were subjected to statistical analysis to calculate the Kappa consistency coefficient and the corresponding P-value, so as to determine the consistency of their assessment results (25). The results showed a Kappa value of 0.85 [95% confidence interval (CI): 0.80 - 0.93] with P < 0.001. According to the Kappa consistency criteria, a value ranging from 0.81 to 1.00 indicates “almost perfect agreement,” which demonstrates that the two researchers’ assessment results were highly consistent.

Given that the sample size of this study reached 814 cases, the normality of continuous variables was assessed using the Kolmogorov-Smirnov normality test. Results indicated that all continuous variables in this study did not conform to a normal distribution, as detailed in **Supplementary Table 1**. Thus, continuous variables were expressed as median (25th - 75th percentile). Differences between groups were evaluated using the Wilcoxon rank-sum test, with U statistics and P values reported. In addition, categorical variables were expressed as frequencies and constituent ratios. Differences between groups were compared using the chi-square test, with χ² values and P values reported.

After that, receiver operating characteristic (ROC) analysis was used to evaluate the predictive value of peripheral TT3, FT3, TT4, FT4, and TSH for the long-term psychological prognosis of patients, with the corresponding area under the curve (AUC), P values, cutoff values, sensitivities, and specificities reported.

Kaplan-Meier survival analysis was used to evaluate the prognostic differences among sepsis patients with different thyroid function statuses, with the log-rank test statistic (χ²) and P values reported.

Multivariate COX regression analysis was used to evaluate the association of the thyroid function with the long-term psychological prognosis of sepsis patients, and hazard rate (HR), 95%CI and P value were reported. In this multivariate model, demographic data (except age), personal history, disease history, and clinical parameters were adjusted. Then, subgroup analysis was conducted based on age to evaluate the association mentioned above.

Spearman rank correlation analysis was adopted to measure the monotonic relationship between thyroid hormones and peripheral inflammatory factors, with Spearman’s rank correlation coefficients (r_s_) and P values reported.

All these analyses mentioned above, a P value less than 0.05 indicated that the difference or association was statistically significant. All analyses were conducted using SPSS 23.0.

Additionally, GPowerWin 3.1.9.7 was used to conduct a *post-hoc* power analysis, aiming to verify whether the existing sample size is sufficient to support the ideal statistical power required for this study.

## 3 Results

### 3.1 Subjects

At baseline, 835 patients who met the inclusion criteria were initially enrolled in this study. During the follow-up period, 21 patients were excluded due to loss to follow-up, refusal to participate, and death. Eventually, 814 patients (97.5%) completed the entire study.

According to the psychological prognosis assessment at the end of follow-up, a total of 261 patients who had at least one of the above-mentioned psychological disorders were classified into the poor psychological prognosis (PPP) group. Another 553 patients, in whom no such psychological disorders were detected, were grouped into the normal psychological prognosis group (NPP).

### 3.2 Basic characteristics of subjects

In **Table 1**, compared with the patients in the NPP group, patients in the PPP group were older than those in the NPP group (P = 0.004). They also had a higher body temperature (P = 0.021), a lower MAP (P = 0.046), higher levels of peripheral white blood cells and lactate (P<0.001, P<0.001), lower levels of peripheral platelets (P<0.001), and higher SOFA scores and SAPS II scores (both P<0.001).

**Table 1:** Baseline characteristics of subjects in the two groups ^a^.

Variables (Baseline)	PPP group (n = 261)	NPP group (n = 553)	U/χ² value	P value b
Demographic data				
Male (n)	143 (54.8)	283 (51.2)	0.928	0.335
Age (years)	67.0 (58.0-75.0)	65.0 (55.0-74.0)	63273.0	0.004
Personal history				
Smoking (n)	68 (26.1)	133 (24.1)	0.383	0.536
Drinking (n)	55 (21.1)	105 (19.0)	0.488	0.485
Disease history				
Hypertension (n)	117 (44.8)	222 (40.1)	1.600	0.206
CHD (n)	63 (24.1)	110 (19.9)	1.910	0.167
T2DM (n)	44 (16.9)	75 (13.6)	1.543	0.214
Clinical parameters				
Heart rate (bpm)	90.0 (79.0-98.0)	89.0 (77.5-99.0)	69926.0	0.474
Temperature (°C)	38.2 (37.1-39.2)	37.9 (36.9-38.9)	64928.5	0.021
MAP (mmHg)	78.0 (69.0-87.0)	79.0 (71.0-88.0)	65912.5	0.046
WBC (×10^9^/l)	12.7 (9.8-15.0)	11.0 (8.5-13.5)	52304.0	<0.001
Haemoglobin (g/l)	95.2 (85.7-102.9)	95.7 (88.4-103.1)	69337.0	0.366
Platelet (×10^9^/l)	188.9 (144.7-236.2)	220.2 (168.8-271.8)	51542.0	<0.001
Creatinine (μmol/l)	67.0 (55.7-82.2)	69.9 (58.0-82.3)	67381.5	0.126
Lactate (nmol/l)	2.9 (2.0-3.8)	2.5 (1.6-3.6)	60812.5	<0.001
SOFA	7.0 (6.0-8.0)	6.0 (4.0-8.0)	51017.0	<0.001
SAPS II	50.0 (40.0-61.0)	43.0 (32.0-53.0)	52109.0	<0.001
Thyroid function				
TT3 (nmol/l)	2.2 (2.0-2.3)	2.3 (2.1-2.5)	54151.5	<0.001
FT3 (pmol/l)	3.4 (2.4-4.4)	4.5 (3.5-5.4)	41966.0	<0.001
TT4 (nmol/l)	101.6 (77.8-115.4)	113.1 (93.2-127.8)	53738.0	<0.001
FT4 (pmol/l)	16.2 (12.5-18.3)	19.5 (15.7-23.0)	46210.5	<0.001
TSH (mU/l)	2.1 (1.0-3.7)	2.2 (1.4-3.2)	70766.0	0.655
Strict-normal (n)	61 (23.4)	190 (34.4)	10.035	0.002
Low-normal (n)	94 (36.0)	226 (40.9)	1.750	0.186
Hypothyroidism (n)	106 (40.6)	137 (24.8)	21.243	<0.001
Peripheral inflammation				
TNF-α (pg/ml)	317.7 (218.4-399.3)	168.2 (92.6-231.7)	24533.5	<0.001
Interleukin-6 (pg/ml)	529.3 (305.3-808.4)	373.3 (187.1-544.8)	45353.0	<0.001
Interleukin-8 (pg/ml)	614.0 (369.0-826.7)	366.5 (198.4-541.7)	35945.0	<0.001

a PPP, Poor psychological prognosis; NPP, Normal psychological prognosis, MAP, Mean arterial pressure; WBC, White blood cell; SOFA, Sequential organ failure assessment; SAPS II, Simplified acute physiology score II, CHD, Coronary heart disease; T2DM, Type 2 diabetes mellitus; TT3, Total triiodothyronine; FT3, Free triiodothyronine; TT4, Total thyroxine; FT4, Free thyroxine; TSH, Thyroid stimulating hormone; TNF-α, Tumor necrosis factor-α.

b The continuous variables were expressed as median (25th - 75th percentile); the categorical variables were expressed by frequency and composition ratio; a P value less than 0.05 indicated that the difference was statistically significant.

The levels of peripheral TT3, FT3, TT4, and FT4 in the patients of the PPP group were all lower than those in the NPP group (all P<0.001). The proportion of patients with strict-normal thyroid function in the PPP group was lower than that in the NPP group (P = 0.002), while the proportion of patients with hypothyroidism in the PPP group was higher than that in the NPP group (P<0.001).

In addition, the levels of peripheral TNF-α, interleukin-6, and interleukin-8 in the patients of the PPP group were all higher than those in the NPP group (all P<0.001).

### 3.3 Psychological prognosis of subjects during the 28-week follow-up

In **Table 2**, there were 149 patients (18.3%), 138 patients (17.0%), and 99 patients (12.2%) who were identified as having anxiety, depression, and PTSD respectively during the follow-up period. It was quite common that the same patient suffered from multiple psychological disorders.

**Table 2:** Psychological prognosis of subjects during the 28-week follow-up ^a^.

Variables (After follow-up)	Criterion	No. of the subjects ^b^ (n = 814)
Anxiety		
Absent (n)	HADS 0 to 7	665 (81.7)
Mild (n)	HADS 8 to 10	97 (11.9)
Moderate (n)	HADS 11 to 14	46 (5.7)
Severe (n)	HADS 15 to 21	6 (0.7)
Depression		
Absent (n)	HADS 0 to 7	676 (83.0)
Mild (n)	HADS 8 to 10	89 (10.9)
Moderate (n)	HADS 11 to 14	42 (5.2)
Severe (n)	HADS 15 to 21	7 (0.9)
PTSD		
Absent (n)	CAPS 0 - 49	715 (87.8)
Mild (n)	CAPS 50 - 60	61 (7.5)
Moderate (n)	CAPS 61 - 100	27 (3.3)
Severe (n)	CAPS 101 - 130	7 (0.9)
Extremely severe (n)	CAPS 131 - 192	4 (0.5)
Total		
NPP group (n)	None of the three	553 (67.9)
PPP group (n)	At least one of the three	261 (32.1)

a HADS, Hospital anxiety and depression scale; PTSD, Post-traumatic stress disorder; CAPS, Clinician-administered PTSD scale; PPP, Poor psychological prognosis; NPP, Normal psychological prognosis.

b The categorical variables were expressed by frequency and composition ratio.

In addition, the above-mentioned evaluations were also carried out at the beginning of the follow-up. The results showed that none of the patients had any psychological disorders, which was also in line with the inclusion criteria of the subjects in this study.

### 3.4 ROC analysis for thyroid function predicting psychological prognosis

**Figure 1:**
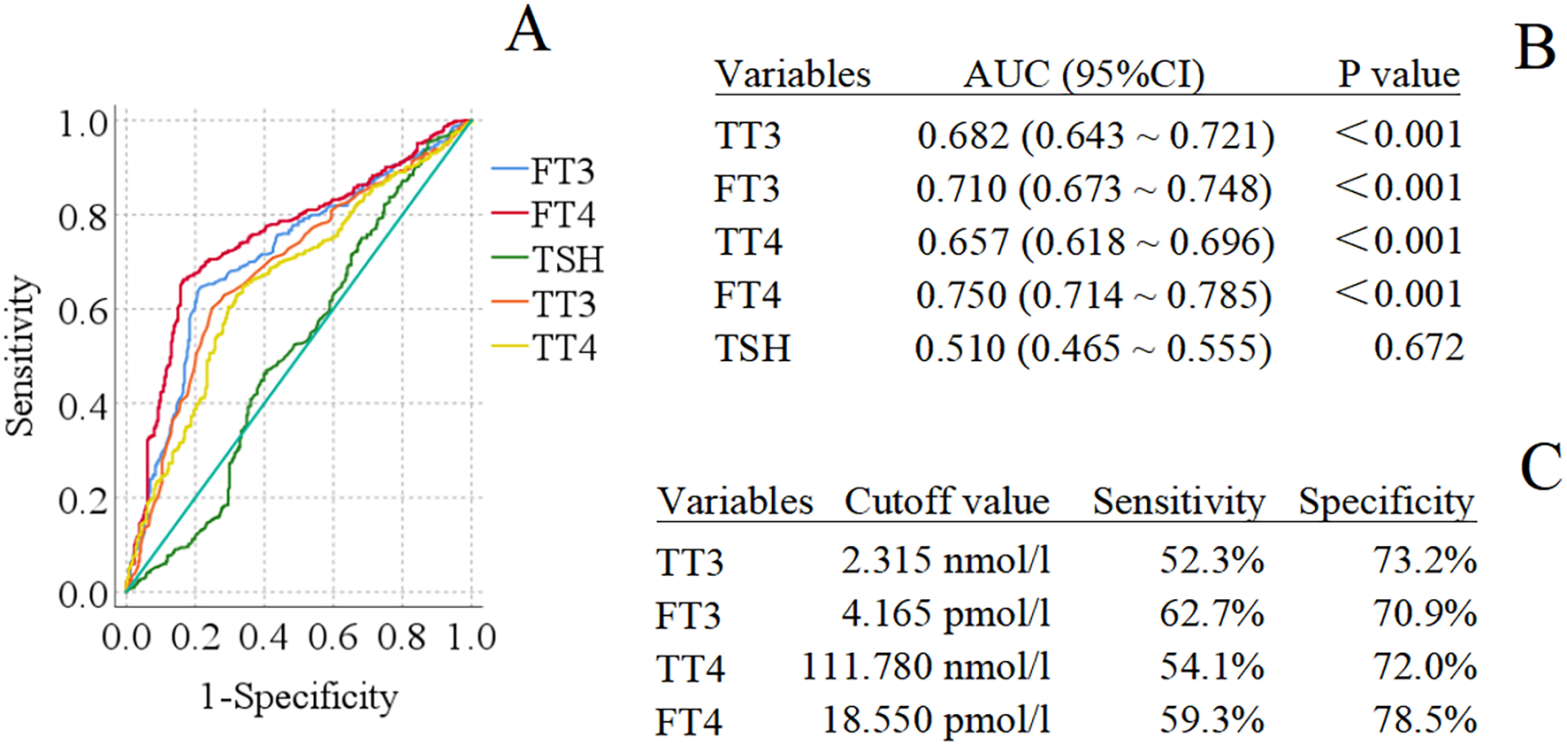
Receiver operating characteristic (ROC) analysis for thyroid function predicting 28-week poor psychological prognosis ^a^. ^a^ TT3, Total triiodothyronine; FT3, Free triiodothyronine; TT4, Total thyroxine; FT4, Free thyroxine; TSH, Thyroid stimulating hormone; AUC, Area under curve; 95%CI, 95% Confidence interval. **(A)** displays the ROC curves of TT3, FT3,TT4, FT4 and TSH for predicting 28-week poor psychological prognosis in sepsis patients; **(B)** shows the detailed data of the TT3, FT3, TT4, FT4 and TSH, including AUC and P values; and **(C)** provides the detailed data of the TT3, FT3, TT4 and FT4, including cutoff values, sensitivities, and specificities.

In **Figures 1A–C**, the four thyroid indicators (peripheral TT3, FT3, TT4, and FT4) showed certain predictive value for poor psychological prognosis within 28 weeks (all P < 0.001). Among them, FT4 had the largest AUC of 0.750, with a cutoff value of 18.550 pmol/l, a sensitivity of 59.3%, and a specificity of 78.5%, thus demonstrating the highest predictive value for poor psychological prognosis within 28 weeks. The cutoff values, sensitivities, and specificities of the other three thyroid indicators are listed in **Figure 1C**. Notably, the sensitivities of these four thyroid indicators were less optimal compared to their specificities. Additionally, the predictive value of TSH was not validated, so no relevant cutoff value was determined.

### 3.5 Kaplan-Meier survival analysis for thyroid function predicting psychological prognosis

According to the cutoff values of peripheral TT3, FT3, TT4, and FT4, all patients were divided into high-level and low-level subgroups based on the cutoff values of these four indicators, respectively. Peripheral TSH was not included in the following analysis.

**Figure 2:**
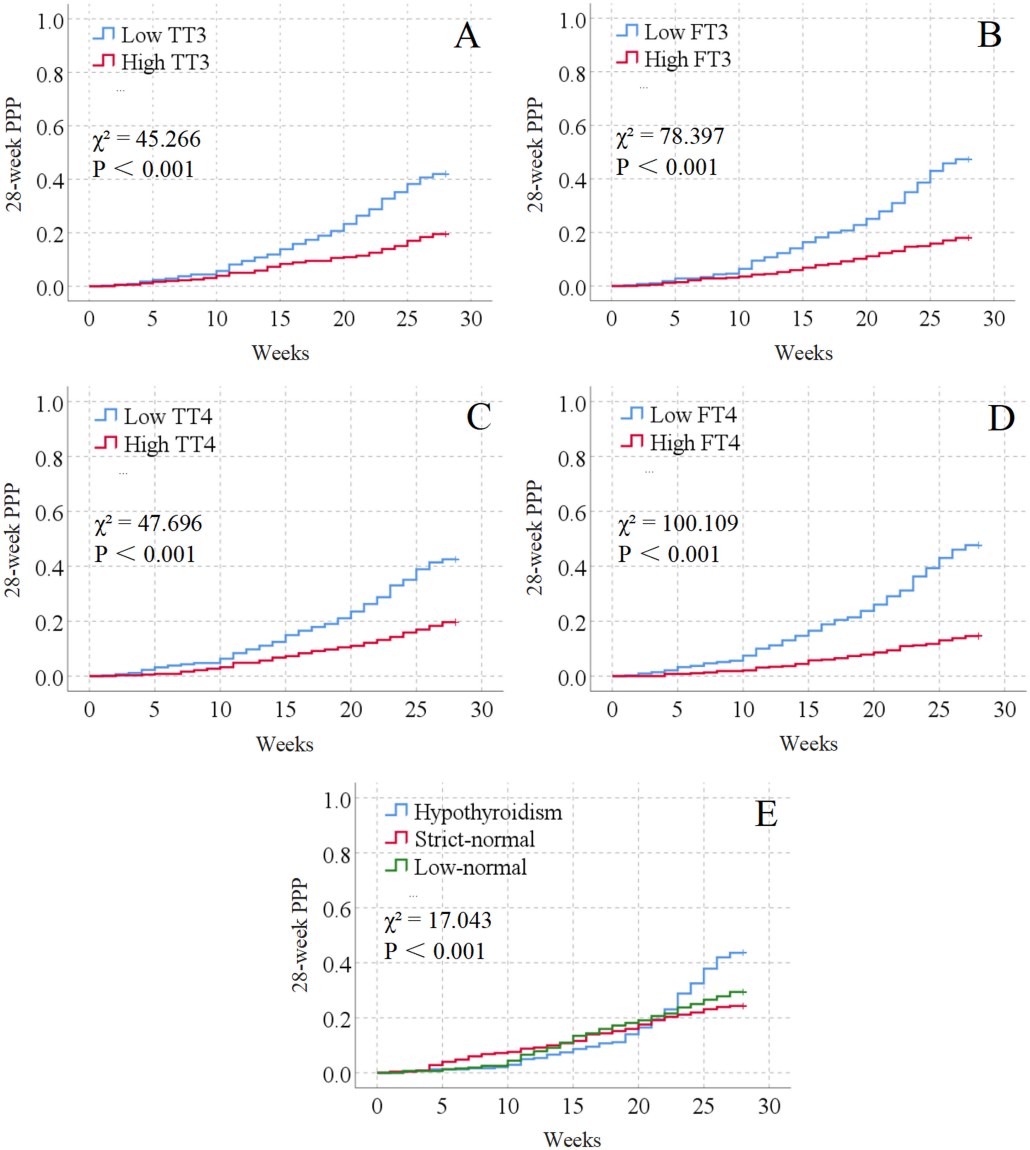
Kaplan-Meier survival analysis for thyroid function predicting 28-week poor psychological prognosis ^a^. ^a^ TT3, Total triiodothyronine; FT3, Free triiodothyronine; TT4, Total thyroxine; FT4, Free thyroxine; PPP, Poor psychological prognosis. **(A–D)** exhibit respectively the Kaplan-Meier curves of 28-week psychological prognosis in patients with different levels (low level vs. high level) of TT3, FT3, TT4, and FT4; **(E)** presents the Kaplan-Meier curves of 28-week psychological prognosis in patients with different thyroid function statuses, namely strict-normal thyroid function, low-normal thyroid function, and hypothyroidism.

In **Figures 2A–D**, compared with the respective high-level groups, the patients with low TT3, FT3, TT4 or FT4 had higher psychological disorder rate within 28 weeks (all P<0.001).

In **Figure 2E**, the patients with strict-normal thyroid function, low-normal thyroid function, or hypothyroidism showed significantly different psychological disorder rates within 28 weeks (P<0.001). Among them, the patients with hypothyroidism had the highest psychological disorder rate within 28 weeks, while the patients with strict-normal thyroid function had the lowest psychological disorder rate (P<0.001).

### 3.6 Multivariate COX regression analysis for thyroid function predicting psychological prognosis

In **Table 3**, after adjusting for several baseline characteristics of the subjects (except age), the multivariate model reported that the high TT3, FT3, TT4, and FT4 on admission were associated with the decreased risk of poor psychological prognosis within 28 weeks (all P<0.001). The multivariate model also reported that the strict-normal thyroid function was associated with the decreased risk of poor psychological prognosis within 28 weeks (P = 0.003), and the hypothyroidism was associated with the increased risk of poor psychological prognosis within 28 weeks (P<0.001).

**Table 3:** Multivariate COX regression analysis for baseline thyroid function predicting 28-week poor psychological prognosis ^a^.

Thyroid variables	Univariate model		Multivariate model ^b^	
(Baseline)	P value	HR (95%CI)	P value	HR (95%CI)
High TT3	<0.001	0.406 (0.309 ~ 0.534)	<0.001	0.473 (0.357 ~ 0.627)
High FT3	<0.001	0.321 (0.246 ~ 0.420)	<0.001	0.371 (0.283 ~ 0.487)
High TT4	<0.001	0.403 (0.308 ~ 0.529)	<0.001	0.440 (0.333 ~ 0.581)
High FT4	<0.001	0.250 (0.186 ~ 0.337)	<0.001	0.286 (0.211 ~ 0.387)
Strict-normal	0.007	0.673 (0.506 ~ 0.897)	0.003	0.645 (0.483 ~ 0.861)
Low-normal	0.275	0.869 (0.675 ~ 1.119)	0.491	0.915 (0.709 ~ 1.179)
Hypothyroidism	<0.001	1.634 (1.276 ~ 2.093)	<0.001	1.582 (1.235 ~ 2.027)

a TT3, Total triiodothyronine; FT3, Free triiodothyronine; TT4, Total thyroxine; FT4, Free thyroxine; HR, Hazard rate; 95%CI, 95% Confidence interval.

b The multivariate model adjusted for demographic data (except age), personal history, disease history, and clinical parameters. A P value less than 0.05 indicated that the association was statistically significant.

Given that age is significantly associated with both sepsis prognosis and thyroid function (26, 27), patients were divided into a middle-aged (<60 years old) and an elderly (≥60 years old) group, and the prior analysis was replicated (**Table 4**). In the middle-aged, peripheral TT3, FT3, TT4, and FT4 related to the risk of psychological prognosis within 28 weeks (P = 0.001, P<0.001, P = 0.009, P = 0.001), yet strict-normal thyroid function and hypothyroidism didn’t (P>0.05). In the elderly, peripheral TT3, FT3, TT4, FT4, strict-normal thyroid function, and hypothyroidism still correlated with the risk within 28 weeks (P<0.001, P<0.001, P<0.001, P<0.001, P = 0.010, P<0.001).

**Table 4:** Multivariate COX regression analysis for thyroid function predicting 28-week poor psychological prognosis (stratified according to age) ^a^.

Thyroid variables	Multivariate model b	
	P value	HR (95%CI)
Age<60 years		
High TT3	0.001	0.442 (0.268 ~ 0.731)
High FT3	<0.001	0.341 (0.205 ~ 0.568)
High TT4	0.009	0.530 (0.329 ~ 0.856)
High FT4	0.001	0.428 (0.256 ~ 0.715)
Strict-normal	0.134	0.659 (0.381 ~ 1.137)
Low-normal	0.604	1.133 (0.707 ~ 1.814)
Hypothyroidism	0.315	1.270 (0.797 ~ 2.024)
Age≥60 years		
High TT3	<0.001	0.436 (0.313 ~ 0.606)
High FT3	<0.001	0.353 (0.257 ~ 0.485)
High TT4	<0.001	0.357 (0.256 ~ 0.498)
High FT4	<0.001	0.218 (0.151 ~ 0.314)
Strict-normal	0.010	0.639 (0.453 ~ 0.900)
Low-normal	0.262	0.840 (0.621 ~ 1.138)
Hypothyroidism	<0.001	1.736 (1.295 ~ 2.327)

a TT3, Total triiodothyronine; FT3, Free triiodothyronine; TT4, Total thyroxine; FT4, Free thyroxine; HR, Hazard rate; 95%CI, 95% Confidence interval.

b The multivariate model adjusted for demographic data (except age), personal history, disease history, and clinical parameters. A P value less than 0.05 indicated that the association was statistically significant.

### 3.7 Monotonic relationship between thyroid hormones and peripheral inflammatory factors

In **Table 5**, the peripheral TT3 levels had a negative relationship with the levels of peripheral TNF-α (P<0.001). Meanwhile, the peripheral FT3 levels had a negative relationship with the levels of all these three peripheral inflammatory factors (all P<0.001). The peripheral TT4 levels had a negative relationship with the level of peripheral TNF-α (P<0.001). And the peripheral FT4 levels had a negative relationship with the levels of all these three peripheral inflammatory factors (P<0.001, P = 0.010, P = 0.001).

**Table 5:** Monotonic relationship between thyroid hormones and peripheral inflammatory factors at baseline ^a^.

Thyroid hormones (Baseline)	Peripheral inflammation (Baseline)	r_s_ value	P value
Peripheral TT3 level	TNF-α level	-0.158	<0.001
	Interleukin-6 level	-0.067	0.056
	Interleukin-8 level	-0.059	0.091
Peripheral FT3 level	TNF-α level	-0.208	<0.001
	Interleukin-6 level	-0.118	<0.001
	Interleukin-8 level	-0.133	<0.001
Peripheral TT4 level	TNF-α level	-0.133	<0.001
	Interleukin-6 level	-0.060	0.085
	Interleukin-8 level	-0.062	0.079
Peripheral FT4 level	TNF-α level	-0.203	<0.001
	Interleukin-6 level	-0.091	0.010
	Interleukin-8 level	-0.114	0.001

a TT3, Total triiodothyronine; FT3, Free triiodothyronine; TT4, Total thyroxine; FT4, Free thyroxine; TNF-α, Tumor necrosis factor-α. A P value less than 0.05 indicated that the relationship was statistically significant.

### 3.8 *Post-hoc* sample size test

*Post-hoc* sample size analysis was performed using GPowerWin 3.1.9.7 software to verify whether the current sample size was sufficient to support the statistical reliability of the core findings. Given that baseline high-level FT4 exhibited the most critical predictive value for poor psychological prognosis within 28 weeks in sepsis patients in the multivariate COX regression analysis (HR = 0.286, 95%CI: 0.211-0.387, P<0.001), this indicator was selected as the representative outcome for analysis.

First, the HR corresponding to FT4 was converted to a point-biserial correlation coefficient, resulting in an effect size of 0.567, which was classified as a large effect size according to Cohen’s criteria. Under the conditions of α=0.05 and 1-β=0.95, the minimum total sample size required was 25 cases. The actual sample size enrolled in this study was 814 cases, which was far larger than the minimum required sample size. This indicates that the current sample size has sufficient statistical power, ensuring the statistical significance and reliability of the results.

## 4 Discussion

At present, thyroid dysfunction caused by sepsis is mainly manifested as a decrease in T3 and T4 levels, while the TSH level may also alter to varying degrees. This is known as low T3 syndrome, which is considered an adaptive response of the body to severe diseases (28, 29). The thyroid function levels in this study were in line with the characteristics of low T3 syndrome. Moreover, none of the subjects in this study had any thyroid diseases before suffering from sepsis. All of these facts confirmed that the thyroid dysfunction of the included participants was due to sepsis, and the data were reliable.

According to the results, this study confirmed that lower baseline levels of T3 and T4 were associated with a poorer psychological prognosis of sepsis; hypothyroidism was also related to a worse psychological prognosis of the disease. These results had adjusted for most potential confounding factors (except age) and possessed a certain degree of independence. In this study, the thyroid function-related factors were divided into continuous variables (such as T3 and T4) and categorical variables (such as hypothyroidism), and the results of these two parts were basically consistent, which proved that these results had a certain degree of stability.

Meanwhile, the ROC curve analysis confirmed that among all thyroid indicators, FT4 exhibited the strongest predictive effect on poor psychological prognosis within 28 weeks after sepsis (AUC = 0.750), which is closely associated with its core role in regulating neurometabolism and inflammatory responses. All four thyroid indicators showed the characteristic of “lower sensitivity than specificity”; this may be attributed to the fact that psychological abnormalities after sepsis are influenced by multiple factors such as inflammation and social support, making it difficult for a single thyroid indicator to cover all cases. However, the high specificity can still effectively reduce false positives, providing a reference for the clinical accurate screening of high-risk populations.

Previous studies had discovered that “low-normal thyroid function”, which was defined as a relatively high level of peripheral TSH within the normal reference range, might lead to negative health consequences similar to those of overt and subclinical hypothyroidism (30, 31). Hence, the present study also investigated the association between “low-normal thyroid function” and the psychological prognosis of sepsis, but no significantly meaningful results were uncovered. A possible explanation was that the impact of “low-normal thyroid function” may be relatively mild and only manifest in certain chronic diseases or age-related disorders.

Additionally, age was a crucial factor affecting sepsis prognosis and thyroid function (26, 27). Thus, this study carried out a subgroup analysis to separately investigate the association between thyroid function and sepsis psychological prognosis in middle-aged and elderly groups. The results showed that in the elderly, the predictive role of thyroid function was highly significant; in the middle-aged, thyroid-related continuous variables had a predictive impact, yet surprisingly, categorical variables had no predictive effect. The explanations were: first, old age might be a potential mediating factor for thyroid function in predicting sepsis prognosis. Second, the cutoff values of T3 and T4 from ROC analysis didn’t fully align with the medical normal value ranges of these indicators, causing differences in predictive significance between thyroid-related continuous and categorical variables. Third, subgroup analysis led to a smaller sample size and reduced test power, more prominent in the middle-aged group.

The preliminary mechanism analysis of this study showed that the levels of several major peripheral inflammatory factors were negatively correlated with the levels of thyroid hormones, especially free thyroid hormones. It is well known that TNF-α, interleukin-6, and interleukin-8 are important inflammatory factors involved in the occurrence and development of sepsis, and thyroid hormones have a biological role in regulating the peripheral inflammation to a certain extent (32–34). Therefore, combined with previous knowledge and the results of this study, it is reasonable to infer that the regulation of some key inflammatory factors may be a potential mechanism by which thyroid hormones affected the degree of sepsis, and may further be the explanation for the predictive role in psychological prognosis.

This study has two limitations. First, although the multivariate analysis adjusted for demographic data (e.g., gender), personal history (including smoking and drinking history), disease history (including hypertension, CHD, and T2DM), and a series of clinical parameters, and additional subgroup analyses were performed based on age, other potential confounding factors remain unaccounted for. These include comorbidities that may contribute to psychological disorders (e.g., chronic obstructive pulmonary disease, chronic liver or kidney dysfunction, or autoimmune diseases), other mental health-related personal histories or habits (such as sleep patterns and physical activity levels), and post-discharge social support. These factors could influence patients’ psychological status through various mechanisms, potentially confounding the results of this study. Second, the follow-up duration was only 28 weeks, with psychological assessments conducted at only two time points: at discharge and week 28. This design fails to dynamically capture fluctuations in psychological issues and hinders analysis of the temporal association between thyroid function and the progression of psychological symptoms. Both limitations are primarily attributed to insufficient medical record data and poor patient compliance. Future studies should adopt more optimized designs to ensure data comprehensiveness, improve patient compliance, and further refine and validate the findings of this research.

In conclusion, this study suggests that a decrease in baseline thyroid function may be associated with an increased risk of anxiety, depression, or PTSD within 28 weeks following sepsis recovery, with this association potentially being more apparent in elderly patients. Peripheral TT3, FT3, TT4, and FT4 could serve as potential predictive indicators for these psychological complications in sepsis patients. However, peripheral TSH and low-normal thyroid function appear to have limited prognostic value in such critical conditions. These findings require further validation in larger, more diverse cohorts.

## Data Availability

The datasets presented in this article are not readily available because not all participants consented to have their data shared. Requests for the anonymized datasets should be directed to the corresponding author.

## Supplementary material

Supplementary Table 1 Results of Kolmogorov-Smirnov normality test ^a^. ^a^ MAP, Mean arterial pressure; WBC, White blood cell; SOFA, Sequential organ failure assessment; SAPS II, Simplified acute physiology score II; TT3, Total triiodothyronine; FT3, Free triiodothyronine; TT4, Total thyroxine; FT4, Free thyroxine; TSH, Thyroid stimulating hormone; TNF-α, Tumor necrosis factor-α. ^b^ A P value greater than 0.05 indicated that the continuous variable conformed to a normal distribution.
