# Supplementary material for: Role of thyroid dysfunction in long-term psychological prognosis of sepsis
Source: Front Psychiatry. 2025 Dec 17;16:1699248. doi: 10.3389/fpsyt.2025.1699248 (PMC12753500; doi:10.3389/fpsyt.2025.1699248)
Supplement: Supplementary Table 1 — Results of Kolmogorov-Smirnov normality test a. a MAP, Mean arterial pressure; WBC, White blood cell; SOFA, Sequential organ failure assessment; SAPS II, Simplified acute physiology score II; TT3, Total triiodothyronine; FT3, Free triiodothyronine; TT4, Total thyroxine; FT4, Free thyroxine; TSH, Thyroid stimulating hormone; TNF-α, Tumor necrosis factor-α. b A P value greater than 0.05 indicated that the continuous variable conformed to a normal distribution. [file SupplementaryFile1.docx]

**Supplemental Table 1 Results of Kolmogorov-Smirnov normality test ^a^**

| **Continuous variables** | **Z value** | **P value ^b^** |
| --- | --- | --- |
| Age | 0.075 | ＜0.001 |
| Heart rate | 0.076 | ＜0.001 |
| Temperature | 0.081 | ＜0.001 |
| MAP | 0.091 | ＜0.001 |
| WBC | 0.079 | ＜0.001 |
| Haemoglobin | 0.072 | ＜0.001 |
| Platelet | 0.061 | ＜0.001 |
| Creatinine | 0.076 | ＜0.001 |
| Lactate | 0.073 | ＜0.001 |
| SOFA | 0.133 | ＜0.001 |
| SAPS II | 0.066 | ＜0.001 |
| TT3 | 0.084 | ＜0.001 |
| FT3 | 0.054 | ＜0.001 |
| TT4 | 0.073 | ＜0.001 |
| FT4 | 0.064 | ＜0.001 |
| TSH | 0.050 | ＜0.001 |
| TNF-α | 0.046 | ＜0.001 |
| Interleukin-6 | 0.067 | ＜0.001 |
| Interleukin-8 | 0.058 | ＜0.001 |

^a^ MAP = Mean arterial pressure, WBC = White blood cell, SOFA = Sequential organ failure assessment, SAPS II = Simplified acute physiology score II, TT3 = Total triiodothyronine, FT3 = Free triiodothyronine, TT4 = Total thyroxine, FT4 = Free thyroxine, TSH = Thyroid stimulating hormone, TNF-α = Tumor necrosis factor-α.

^b^ A P value greater than 0.05 indicated that the continuous variable conformed to a normal distribution.
